# A deep explainable artificial intelligent framework for neurological disorders discrimination

**DOI:** 10.1038/s41598-021-88919-9

**Published:** 2021-05-05

**Authors:** Soroosh Shahtalebi, S. Farokh Atashzar, Rajni V. Patel, Mandar S. Jog, Arash Mohammadi

**Affiliations:** 1 Concordia Institute for Information Systems Engineering, Concordia University, Montreal, QC H3G 1M8 Canada; 2 Departments of Electrical and Computer Engineering, and Mechanical and Aerospace Engineering, New York University (NYU), New York, NY 10003 USA; 3 NYU WIRELESS and NYU Center for Urban Science and Progress (CUSP), New York University (NYU), New York, NY 10003 USA; 4 Department of Electrical and Computer Engineering, Western University, London, ON N6A 5B9 Canada; 5 Department of Clinical Neurological Sciences, Western University, London, ON N6A 3K7 Canada

**Keywords:** Movement disorders, Computational science

## Abstract

Pathological hand tremor (PHT) is a common symptom of Parkinson’s disease (PD) and essential tremor (ET), which affects manual targeting, motor coordination, and movement kinetics. Effective treatment and management of the symptoms relies on the correct and in-time diagnosis of the affected individuals, where the characteristics of PHT serve as an imperative metric for this purpose. Due to the overlapping features of the corresponding symptoms, however, a high level of expertise and specialized diagnostic methodologies are required to correctly distinguish PD from ET. In this work, we propose the data-driven $\text {NeurDNet}$ model, which processes the kinematics of the hand in the affected individuals and classifies the patients into PD or ET. $\text {NeurDNet}$ is trained over 90 hours of hand motion signals consisting of 250 tremor assessments from 81 patients, recorded at the London Movement Disorders Centre, ON, Canada. The $\text {NeurDNet}$ outperforms its state-of-the-art counterparts achieving exceptional differential diagnosis accuracy of $95.55\%$. In addition, using the explainability and interpretability measures for machine learning models, clinically viable and statistically significant insights on how the data-driven model discriminates between the two groups of patients are achieved.

## Introduction

The population of seniors (aged 60 and above) is estimated to rise from 962 million in 2017 to 2.1 billion by 2050, and 3.1 billion by 2100^1^, which proportionally increases the population of the individuals affected by neurological movement disorders^2^. To better manage the growing population of patients, specialized and advanced technologies are required to prevent, control, and cure neurological diseases. Parkinson’s disease (PD) and essential tremor (ET) are among the common neurological movement disorders, which respectively occur at the prevalence rate of $\sim 2\%$ and $\sim 4.5\%$ for individuals over 65 years of age^3,4^. PD and ET share some common symptoms, including pathological hand tremor (PHT), which affects coordination, targeting, and speed of voluntary motions^5^ by the involuntary and pseudo-rhythmic movement of limbs^6^. There are various categorizations of PHT but two types are very common, namely “Rest Tremor” and “Action Tremor”, where the latter is further classified into three subcategories of postural, kinetic, and isometric tremors^7,8^. Rest tremor occurs when a limb is in a resting state and is supported against gravity, while action tremor occurs in case of voluntary contraction of muscles in a limb. Postural, kinetic, and isometric tremors are respectively observed when a patient maintains a position against gravity (such as stretched-out arms), performing a voluntary action, and contraction of muscles against a rigid object. While both PD and ET patients develop tremors, there are characteristic differences, potentially allowing differentiation of these two diseases. More specifically, PD is typically characterized by unilateral rest tremor in the spectral range of 4–6 Hz^9^, whereas ET patients commonly show symmetric postural and kinetic tremor in the range of 4–8 Hz^9^.

Although PD and ET could be characterized by the type of their tremor, they also share overlapping features, especially in the early stages of the diseases^10^. For instance, both rest and action tremors are observed in PD and ET patients to the extent that 46% of ET population show rest tremors^11^ and up to 90% of PD patients have action tremor^8,12,13^. In addition, a considerable number of ET patients show asymmetric hand tremors^8,13,14^, given the fact that asymmetry of PHT is sometimes seen as a key signature of PD. In addition, the age range in which patients start to develop symptoms of PD or ET is not significantly different, further complicating the differential discrimination of the two diseases^15,16^.

The aforementioned overlapping features of PD and ET makes it significantly challenging to conduct differential diagnosis^10,15,17,18^, to the extent that 37% of ET patients are misdiagnosed and most of them are diagnosed as PD. Several studies^17,18^ have shown that 15–35% of patients with other movement disorders are also misdiagnosed as PD. Misdiagnosis of PD and ET can adversely affect the outcome of clinical trials and results in suboptimal treatment and faulty prognosis^10,15^. Consequently, it is of paramount importance to develop and devise advanced diagnosis techniques to significantly avoid such misdiagnosis of PD and ET.

In order to decrease the misdiagnosis rate, in the literature, some sophisticated technological solutions have been proposed to monitor symptoms of patients and track the correlated physiological phenomena. In this regard, recently, Positron emission tomography (PET) has been employed to study brain functions in the case of neuro-degenerative disorders, including PD. Scanning dopamine transporters (DAT) with PET^19,20^ or single photon emission tomography (SPECT) have been recently considered as the gold standard (according to references^21,22^) for differential diagnosis of PD from ET, especially for ambiguous cases^23^. However, due to the expensive and time-consuming nature of PET and SPECT technologies and the need for injecting radioactive-labeled tracers, they are not widely employed^24^ and thus investigating alternative diagnostic procedures is of high importance. In this regard, basic time-series analyses of tremorous motion of the limbs, and electrical activity of muscles are suggested as potential biomarkers that can help with the diagnosis^12,24^. The frequency contents of such recordings are known to reveal useful information for discrimination of PD and ET^7^. Thus, signal processing (SP) and machine learning (ML) techniques are investigated for such analysis of hand motion recordings of patients to better identify and discriminate the underlying characteristics, and assess the associated severity index.

To use time-series recordings for differentiating PD patients from those with similar symptoms but with different diagnosis, several classification schemes are developed over recent years in the literature^25–34^, including statistical signal processing^35^, support vector machines (SVM)^36^, Naive Bayes classifiers, nearest centroid classifier (NCC), random forest (RF)^37^, decision tree (DT), and linear discriminant analysis (LDA)^38,39^. More recently, deep learning (DL) methods, which are considered as a subcategory of ML techniques and present methodologies to design multi-layer artificial neural networks (ANN) are employed to analyze the tremor signals^40,41^. The main benefit of DL methods compared to classical approaches is their independence from expert-defined features to grasp the underlying patterns of data. A meaningful representation of the signals is formed by a DL model when numerous training examples are being observed by the network to minimize a predefined cost function (e.g., classification error). Carefully crafted DL frameworks have shown superior performance in several practical applications and have ignited a great surge of interest in applying them to many different problems. However, the data-hungry nature of the DL techniques demands large datasets, which can represent a broad and clear image of the studied phenomenon and can help the network grasp a generic image of the characteristics of the two diseases from the tremor recordings. In fact, large datasets are required to grant an acceptable degree of the generalization to a neural network^42^ to be securely deployed in real-world applications. Table 1 summarizes the research works on analysis of time-series recordings of tremorous limbs for diagnostic purposes, along with their achieved accuracy.

A growing surge of interest is observed in deploying DL methods, more specifically convolutional neural networks (CNN), in analyzing time-series recordings of tremorous limbs. In CNNs, a number of initially-randomized kernels (filters) are designed and convolved with raw data to capture the underlying patterns^43^. Commonly, several filter layers (hence the term deep learning) are stacked to derive a new informative representation. Technically, CNNs have outperformed computer-level and human-level performance in image^44^ and speech recognition^45^, justifying the growing trend of their application in other fields, e.g., tremor assessment^25,40,41,46–48^. The superior performance of CNN in the analysis of tremor recordings could be contributed to the fact that CNNs, as a subcategory of data-driven ML algorithms, do not require hand-crafted and expert-defined features to understand the studied phenomena and the inference is made by observing a considerable number of training examples and optimizing the parameters of neural network based on minimizing a predefined cost function. One of the main challenges of data-hungry deep neural networks is the interpretability of the results. Although high performance can be achieved, sometimes the network may focus on hidden biases in the dataset. For example, if the signals of the two conditions are recorded using two different machines (with particular spectrotemporal characteristics), a black box neural network may learn how to differentiate between the recording of the two machines, instead of the characteristics of the two conditions. To avoid that, researchers constantly evaluate all possible biases in the dataset, but without an interpretable solution this is always a concern. To address this issue and to encode a degree of transparency and interpretability in the machine learning models, a new set of techniques, referred to as explainable AI or XAI for short, are developed.

In this work, inspired by the significant potentials of XAI and interpretable DL techniques, we propose an innovative DL-based data driven model, referred to as $\text {NeurDNet}$, for accurate and efficient differentiation of Parkinsonian tremor from essential tremor via hand motion recordings. $\text {NeurDNet}$ takes advantage of a 2-stage classification paradigm incorporating a DL core and a classical ML core, to accurately distinguish and classify the recording of patients with PD and ET. $\text {NeurDNet}$ is developed based on a unique, large, and inclusive dataset of hand kinematics, that we clinically collected in this study, which includes 250 tremor assessments of 81 patients. Each tremor assessment consists of recordings in 3 channels from 7 tasks, each recorded in 3 trials. As will be discussed later in “Methods”, the recordings of only six tasks are employed to develop NeurDNet. The collection of recordings from 6 tasks in 3 channels and in 3 trials add up to 54 single-channel tremor signals for each tremor assessment. It is worth highlighting that the 3 trials of data collection during each of the patient visits to the clinic, are mandated by the data collection protocol, and do not imply any sort of decomposition applied to the signals. The utilized dataset was collected at the London Movement Disorders Centre laboratory over a time span of 4 years. The comprehensive employed dataset of hand motion recordings has provided $\text {NeurDNet}$ with the unique capability of perfectly magnifying and mastering the overlapping features of the two disorders (i.e., PD and ET), hence, decreasing the misdiagnosis error and maximizing the classification accuracy. The exceptionally large and inclusive dataset enables $\text {NeurDNet}$ to reliably capture the underlying and overlapping features of the two diseases and provides an acceptable degree of generalization to the network. After publication of this article, we will release the trained $\text {NeurDNet}$ as an open-source library that can be used by other researchers and clinicians. In summary, the paper makes the following key contributions:

- A novel data-driven architecture, i.e., $\text {NeurDNet}$ is developed and trained over a large and comprehensive dataset of hand kinematics collected over a time span of 4 years and consisting of about 90 h of recordings from 81 patients. This dataset has captured the acceleration of hand motion in PD and ET patients in 3-axes, while performing 7 different tasks in 3 trials, by mounting a triaxial accelerometer on the dorsum of their hand.
- The processing pipeline of $\text {NeurDNet}$ is a sequential architecture of a CNN core and a classical ML core, which together form a two-stage classification paradigm for differential diagnosis. This novel architecture further boosts the reliability and accuracy of the system in differentiating Parkinsonian tremor from ET.
- To maximize the amount of extracted information from the dataset with the ultimate goal of maximizing the overall classification accuracy, in addition to the raw accelerometer signals, we introduced shortcut bits to the deep neural architecture of $\text {NeurDNet}$ to convey some information about the task associated with the tremor recording. This is critical, since different tasks would stimulate different characteristics of tremor in PD and ET patients. In other words, we have embedded the label of the tasks performed by each patient as a hint vector in the final classification layer of the neural network to further boost the classification accuracy of $\text {NeurDNet}$ in distinguishing the two diseases. As a result, patients should conduct a particular series of motion tasks (explained later) to activate different PHT patterns, which can be decoded into differential diagnosis using $\text {NeurDNet}$.
- Another major novelty of $\text {NeurDNet}$ is employment of specialized and sophisticated methods in interpreting its decisions by explaining the clues in the input signals that lead to a particular class label. Such comprehensive analysis provides statistically significant and clinically viable knowledge for classification of PD and ET and relaxes the concerns on learning structural and unwanted biases in the input data that can lead to proper discrimination of the two diseases.

The above-mentioned contributions of $\text {NeurDNet}$ collectively have resulted in the state-of-the-art mean classification accuracy of $95.55\%$. Please note that in this paper, by the term“classifying/differentiating PD from ET” we are referring to differentiating between the dynamical behavior of tremor in PD patients with that of ET patients, which can be potentially used as an assistive tool for clinical diagnosis or tremor management.

**Table 1:** Literature review of the recent works in ET/PD classification.

References	Goal	Dataset	Method	Results
Hossen et al.^23^	ET/PD classification	Accelerometer data, [19 PD, 21 ET] for training and [20 PD, 20 ET] for testing	Statistical Signal Characterization performed on the spectral domain of tremor signals	Accuracy = 90%
Ghassemi et al.^31^	ET/PD classification	Electromyogram and accelerometer data, [13 PD, 11 ET] for training and testing	Classification of Wavelet features with Support Vector Machines (SVM)	Accuracy = 83%
Brzan et al.^49^	ET/PD Classificclassificationation	Electromyogram data [27 PD, 27 ET] for training and testing	A set of statistical and physiological features classified with decision tree	Accuracy = 94%
DiBiase et al.^15^	ET/PD classification	Accelerometer data, [16 PD, 20 ET] for training and [55] for testing	Analysis in spectral domain	Accuracy = 92%, Sensitivity = 95%, Specificity = 95%
Barrantes et al.^50^	ET/PD/Healthy classification	Accelerometer data, [17 PD, 16 ET, 12 healthy, 7 unknown]	Spectral analysis of the signals	Accuracy=84.38%
Molparia et al.^51^	ET/PD classification	Accelerometer data and genetic profiles, [40 PD, 27 ET] for training and testing	Statistical properties of signal along with genomics data	Sensitivity = 76%, Specificity = 65%
Locatelli et al.^52^	ET/PD classification	Low power wearable device, [17 PD, 7 ET] for training and testing	Various machine learning techniques	Accuracy=$95.8\%$
Moon et al.^53^	ET/PD classification	Gain and balance characteristics, [524 PD, 43 ET] for training and testing	Hand-crafted features and classical ML	Accuracy =$92\%$
Dugue et al.^54^	ET/PD classification	Accelerometer data, [17 PD, 16 ET, 12 Healthy, 7 inconclusive]	Spectral features and various ML techniques	Accuracy = $84.4\%$

## Methods

In this section, the data collection procedure for the employed dataset as well as the architecture of the $\text {NeurDNet}$ framework and the rationales behind its design are discussed. Lastly, the evaluation metrics and the algorithmic workflow of $\text {NeurDNet}$ are explained.

### Dataset

The dataset employed in this work was collected from 81 PD and ET patients under a single-centre, pilot study approved by the Western University’s Health Sciences Research Ethics Board (HS REB#: 104584 and 107433) at the London Movement Disorders Centre in London, Ontario, Canada. The study protocol is registered with the “www.clinicaltrials.gov” registry (Identification numbers: NCT02551848 and NCT02668497). It is worth highlighting that the NCT02551848 study concerns the data collection from ET patients and the NCT02668497 study examines the characteristics of PHT in PD patients. The data collection procedure and the experiments were performed in compliance with the WMA Declaration of Helsinki, as well as the Tri-Council Policy Statement of Ethical Conduct for research Involving Humans in Canada. The study protocol has received full board approval of the ethics committee and the consent procedure is confirmed as required in the documentation checklist. All the participants in this study were recruited by the Movement Disorder Centre, at the University Hospital, London, Ontario, Canada. They entirely matched the inclusion/exclusion criteria as discussed in References^55,56^, and provided written informed consent for their participation. The first patient’s first tremor assessment was recorded in March 2014, and the last recording occurred in January 2018.

**Figure 1:**
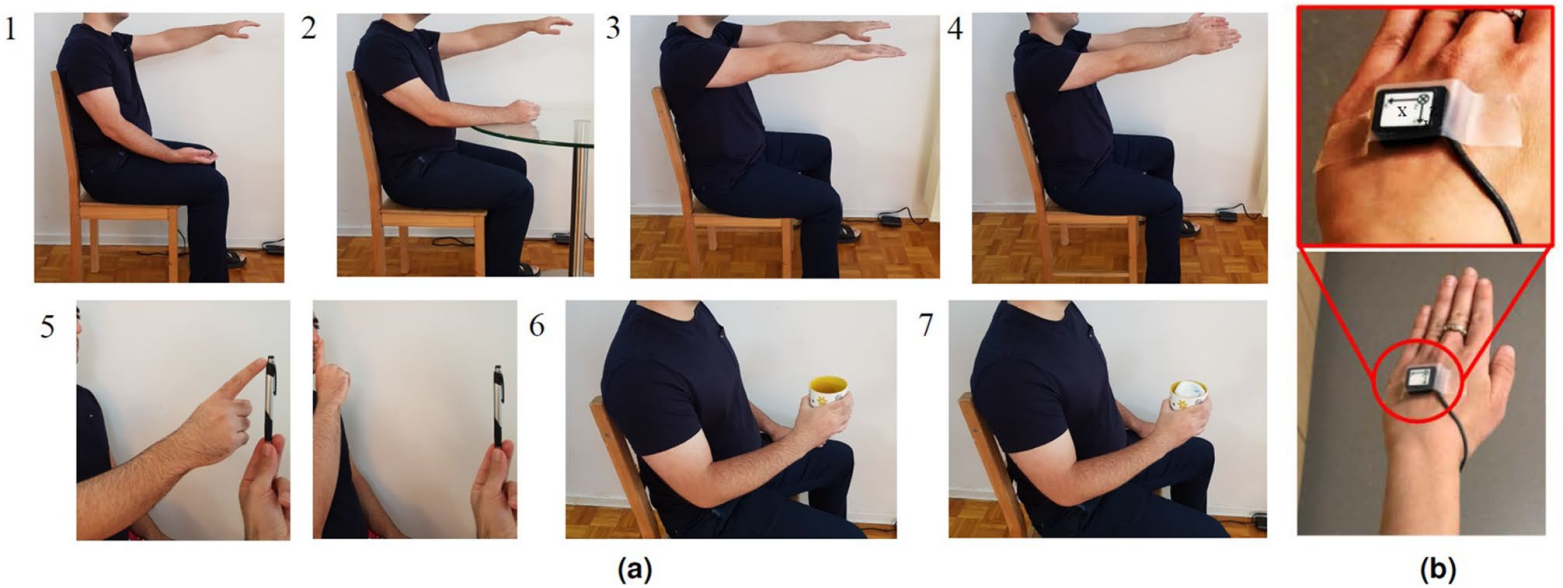
(**a**) Illustration of the 7 scripted tasks performed by PD and ET patients for each tremor assessment. (1) Rest-1; (2) Rest-2; (3) Posture-1; (4) Posture-2; (5) action tremor (repetitive finger to nose motion); (6) Load-1 (empty cup); (7) Load-2 (1-lb weight in the cup). (**b**) Placement of the 3-axis accelerometer sensor on the dorsum of hand. Please note that this figure is reproduced from the Figure 1 of the work by Shahtalebi et al.^25^.

A convenience sampling of 119 PD and 131 ET upper-limb tremor assessments, collectively recorded from 81 patients (47 PD and 34 ET), were employed to develop the $\text {NeurDNet}$ framework. In the PD group, 8 females and 39 males, with the average age of $71.51 \pm 7.63$ years old were recorded, where 14 of them provided bilateral and 33 provided unilateral recordings. In addition, 26 PD patients were de novo, 45 patients have participated twice with a time interval of 6 weeks and only 2 of them have participated once. The ET group contained 34 patients, 13 females and 21 males, with an average age of $69.8 \pm 6.12$ years old. The ET group included 22 de novo patients, where only 3 patients participated for one time and the rest participated for two times with a time interval of 6 weeks. All the ET patients were recorded bilaterally. The analysis of upper-limb kinematics was performed on tremor assessments recorded from patients based on 7 scripted tasks where each one is performed for 20 seconds and the whole process was repeated for 3 times. As previously detailed in References^55,56^ and shown in Fig. 1a, the 7 scripted tasks included two rest positions with the forearm supported on lap (“Rest-1”) or on a table (“Rest-2”), two postural positions with outstretched arms and hands facing the ground (“Posture-1”) or facing each other (“Posture-2”), two weight-bearing tasks with participants holding an empty cup (“Load-1”) or a cup with a 1-lb weight (“Load-2”), and one kinetic task where participants repetitively performed the finger-to-nose action. Thus, 6 of the 7 tasks recorded the PHT in a static position (denoted as “static tremor”) and the finger-to-nose task provided “action tremor” data. As shown in Fig. 1b, an inline 3D accelerometer sensor (#317A Noraxon U.S.A. Inc.) was placed on the dorsum of the hand to capture the PHT in real-time using TeleMyoTM G2 at 1500 Hz and transmit it to a computer running MyoResearch XP Version 1.08.0951.62 software. It is worth noting that the TeleMyoTM G2 device, which is a wireless telemetry system for EMG and inertial sensors, can only provide recordings at either 1500 or 3000 samples/s/channel. The recorded signals include 3 channels of data representing acceleration in the *x*,*y* and *z* axes. In total, 87.5 h of data was employed in this work which were collected from 81 patients (3 channels for each patient, 7 minutes per tremor assessment, and 250 tremor assessments in total).

### Data preparation

Prior to utilizing the recordings for training and evaluation stages, the entire tremor signals were downsampled to 100 Hz to minimize the computational burden on the system as well as the complexity of the network. It should be noted that as the informative spectral region in the tremor signals spans the range up to 20 Hz, and according to the Nyquist theorem that sampling a signal with at least twice the rate of its maximum informative frequency is enough to fully reconstruct it, we believe that 100 Hz is low enough to avoid excessive computational costs on the system and high enough not to distort the spectral contents of interest in the signal. Afterwards, the mean of each signal is subtracted from itself to eliminate the effect of calibration and the bias associated with the posture of each task.

As discussed earlier, the introduced large and inclusive dataset for development of the $\text {NeurDNet}$ framework consists of 250 tremor assessments, where each includes PHT recordings in 3 trials, 7 tasks, and 3 channels. It should be highlighted that to develop and evaluate $\text {NeurDNet}$ framework, we omitted the action tremor recordings associated with the “finger-to-nose” task from the dataset. As the action tremor recordings contain dynamic features from both the person’s voluntary movement and the tremorous movements, we believe that the wide range of characteristics and dynamic properties of the voluntary component misleads $\text {NeurDNet}$ in the classification tasks and degrades its accuracy. There is a general consensus in the literature of PHT processing works^25,57,58^ that the recorded PHT signals can be modeled as the summation of a voluntary and an involuntary component. A major bottleneck in distinguishing between the two components is that no ground truth can be assumed for either of the two components. Since the collected data from the finger-to-nose task represent action tremor signals (voluntary plus involuntary components) where no ground truth can be assumed for their underlying components, we have omitted the recordings of this task from the dataset to mainly focus on the information conveyed through other tasks ,which represent static tremor (i.e., no voluntary motion is present). In other words, by removing this task from our dataset (using 6 tasks to develop the NeurDNet), we have minimized the effect of unknown and untraceable factors on the NeurDNet inference from the recordings.

**Figure 2:**
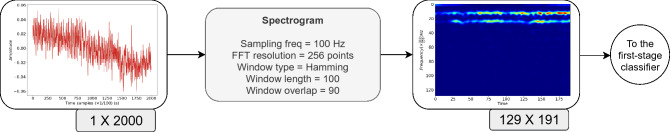
The preprocessing step to convert time-series tremor recordings into 2D spectrotemporal representations of the signals to be processed with the first-stage classifier of $\text {NeurDNet}$.

After removing the recordings of the “finger-to-nose” task, a large collection of 13, 500 ($250 \times 3 \times 6 \times 3 = 13{,}500$) tremor signals constitute the available number of tremor signals for the development of $\text {NeurDNet}$. Finally, for each tremor signal, which is of 20 s length, its spectrogram is calculated according to the Welch method, by sliding a Hamming window of length 100 points, the overlap size of 90 points, and the FFT resolution of 256 points over the tremor signal, which results in a 2-dimensional representation of each signal with the size of $[129 \times 191]$. As shown in Fig. 2, the obtained spectrograms of the tremor signals are then fed to $\text {NeurDNet}$ to be processed by the convolutional layers of the first-stage classifier.

To develop the first-stage classifier and identify the hyper-parameters of neural network, we split the dataset based on [75–25%] portions for training and testing, where the 5-fold cross-validation is performed using the samples in the training set. It is worth noting that the two sets are formed based on subjects and the recordings of one subject only contribute to one set, as an attempt to eliminate any direct or indirect leakage of information from the training set into the test set. Once the hyper-parameters of the first-stage classifier were determined, the second-stage classifier was added to the system and the whole pipeline was trained and evaluated for different training/test proportions. In other words, we have employed [61, 20], [54, 27], [46, 35], [38, 43], [30, 51], and [22, 59] number of patients respectively to form [training, test] sets in $25\%$, $35\%$, $45\%$, $55\%$, $65\%$, and $75\%$ cases. It is also worth mentioning that the whole process of fine-tuning the hyper-parameters of the first-stage classifier is based on the average accuracy of classification in the cross-validation process, and the test set is only employed to perform the final evaluation of $\text {NeurDNet}$, as shown in the results reported in Tables 2 and 3.

**Table 2:** Classification accuracy of $\text {NeurDNet}$ in the two cases of employing binary and probabilistic features.

Classifier	Binary features						Probabilistic features					
	25%	35%	45%	55%	65%	75%	25%	35%	45%	55%	65%	75%
RF (entropy)	85.69	84.24	82.91	81.94	82.43	78.68	86.18	85.43	83.79	82.66	82.20	78.21
RF (gini)	85.43	84.59	83.43	82.35	81.97	78.28	86.49	84.81	84.27	82.63	82.57	78.29
SVM (rbf)	85.68	84.65	84.24	82.19	83.10	79.46	86.33	85.83	85.38	82.09	82.68	79.01
SVM (linear)	84.26	82.69	82.08	81.34	80.78	78.02	85.83	84.77	83.60	82.36	82.02	78.57
NB	83.70	83.55	80.23	81.44	81.67	77.31	85.98	86.42	84.94	83.94	84.15	81.48
LR	85.76	84.41	84.09	83.10	82.83	79.49	**87.29**	86.10	85.28	83.65	83.38	79.74
AdaBoost	83.97	81.61	80.99	79.95	79.30	75.80	85.03	82.97	81.53	80.01	78.12	73.32
LDA (svd)	79.54	76.25	75.83	73.79	66.21	67.44	77.81	76.41	76.56	72.31	65.12	63.62
LDA (lsqr)	79.54	76.25	75.80	73.77	63.40	49.57	77.81	76.41	76.56	72.31	65.12	49.50
QDA	81.85	83.18	78.69	72.08	63.26	58.62	** 95.55**	**93.89**	81.73	73.48	56.29	53.13
DT (entropy)	81.21	78.45	77.66	77.63	76.02	74.75	80.40	79.01	77.11	77.57	75.06	71.73
DT (gini)	80.45	80.16	78.51	77.25	77.32	75.25	77.99	78.29	76.89	76.35	74.29	71.84
MLP (10)	85.01	82.40	82.05	81.25	79.79	77.53	84.33	83.03	81.64	80.25	80.04	77.04
MLP (30)	84.64	82.84	82.02	80.85	79.63	77.49	84.53	82.80	81.79	80.50	80.33	77.45

The classification accuracy is measured across different choices of the second-stage classifier, including random forests (RF), support vector machines (SVM), Naive Bayes Classifier (NB), logistic regression (LR), AdaBoost classifier (AB), linear discriminant analysis (LDA), quadratic discriminant analysis (QDA), decision trees (DT), and multi layer perceptron (MLP).

**Table 3:** Classification accuracy of $\text {NeurDNet}$ when only the first-visit tremor assessments are included in the test set.

Classifier	Binary features						Probabilistic features					
	25%	35%	45%	55%	65%	75%	25%	35%	45%	55%	65%	75%
RF (entropy)	87.31	85.30	83.66	81.90	81.43	79.60	86.78	86.13	84.78	82.36	81.53	81.05
RF (gini)	87.59	85.80	83.50	82.03	80.96	79.77	86.66	85.63	84.83	82.23	81.38	80.83
SVM (rbf)	87.05	85.89	84.51	82.07	81.45	78.66	**88.26**	86.50	86.13	82.81	82.22	79.85
SVM (linear)	85.85	82.56	82.47	81.15	79.81	77.90	86.82	84.86	83.83	82.39	81.34	80.14
NB	84.99	83.93	79.95	81.65	77.07	75.61	87.60	86.44	85.11	84.54	82.57	81.09
LR	87.43	85.26	84.05	81.88	80.92	78.78	88.08	86.62	86.30	83.52	82.74	80.94
AdaBoost	86.26	82.53	82.70	80.21	77.69	76.46	85.79	83.59	82.32	80.78	78.63	75.06
LDA (svd)	81.13	78.10	76.10	70.13	67.04	67.82	79.12	77.02	76.49	71.58	66.14	62.99
LDA (lsqr)	81.13	78.10	76.04	70.13	62.49	49.41	79.12	77.02	76.49	71.58	66.14	51.06
QDA	79.18	80.77	77.65	70.20	62.15	60.04	**93.05**	**89.66**	77.59	71.63	59.92	54.01
DT (entropy)	80.85	79.04	78.25	76.60	76.42	74.96	79.76	79.14	78.44	77.39	75.61	73.82
DT (gini)	81.78	80.40	78.63	76.86	75.54	73.97	80.35	77.90	78.09	77.47	76.28	74.15
MLP (10)	85.41	83.33	82.54	78.90	78.09	77.60	83.31	81.84	81.50	79.72	78.15	77.83
MLP (30)	85.74	82.76	81.48	79.00	78.42	77.23	83.80	82.24	81.87	79.70	78.22	77.55

The classification accuracy is measured across different choices of second-stage classifier, including random forests (RF), support vector machines (SVM), Naive Bayes Classifier(NB), logistic regression (LR), AdaBoost classifier (AB), linear discriminant analysis (LDA), quadratic discriminant analysis (QDA), decision trees (DT), and multi layer perceptron (MLP).

It is worth highlighting that due to the balance of data in the two classes of our dataset, the classification accuracy is utilized as a reliable metric to conclude and compare the performance of NeurDNet across different scenarios. Here, the classification accuracy is derived by dividing the number of correctly classified patients over the total number of patients in the test set. In addition, to obtain the best hyper-parameters for NeurDNet in order to achieve the highest classification accuracy, statistical significance tests are employed to statistically verify the effect of each hyper-parameter on the final classification accuracy. Please note that this process is employed to fine-tune the type of output for the first-stage classifier and the classification paradigm in the second-stage classifier. It should be noted that this process is different from the 10-fold cross validation strategy employed for fine-tuning the hyper-parameters of the first-stage classifier.

### Hyper-parameter optimization of $\text {NeurDNet}$

In the validation process, all of the parameters and hyper-parameters of $\text {NeurDNet}$ are fine-tuned to maximize the classification accuracy. To fine-tune the hyper-parameters of the first-stage classifier, which is a CNN-based deep neural model, the hand motion dataset is strictly split into 2 sets, $75\%$ for training and $25\%$ for testing. To avoid the leakage of information from the training set to the test set, the formation of datasets is based on tremor assessments from patients and the recordings of each patient are only participated in one set. This strategy is used to impose harsh evaluation conditions on $\text {NeurDNet}$ to better investigate its capability in extracting the generic underlying patterns of each disease from the hand motion recordings. To identify the optimum hyper-parameters of the network and validate its performance over different hyper-parameters, we employed a 5-fold cross-validation procedure over the samples in the training set. In fact, each round of training is performed over 4/5 of the training set and the rest of the samples are utilized for validation and this process is repeated for 5 times with completely exclusive validation samples. Finally, the mean performance over the 5 runs is reported as the accuracy of network for the selected hyper-parameters. In addition, cross-validation enables us to decide if the model is overfitted to training samples or not and investigate if the network generalizes well over the wide and overlapping range of hand motion characteristics for the two diseases. It is worth highlighting that the classification accuracy of the first-stage classifier refers to correct classification rate over the input spectrograms (formation of the training and validation sets is based on patients so that tremor signals of one patient only contribute in either the training or the validation set). A rigorous grid-search strategy is adopted to try different potential hyper-parameters for the CNN and they are compared based on the 5-fold cross validation classification accuracy. The pool of hyper-parameters includes the number of convolutional layers, kernel size, the number of dense layers, width of dense layers, optimizer, and the learning rate. To summarize, $75\%$ of data (10, 125 samples) is used for training, $25\%$ (3375 samples) is reserved for evaluation. The best classification accuracy of the first-stage classifier over the validation data is $75.55\%$. It should be noted that each tremor assessment consists of 54 tremor signals ($6\, \text {tasks} \times 3 \,\text {trials} \times 3\, \text {channels}$) and the above-mentioned accuracy is achieved for classification of each tremor signal, therefore, the achieved performance does not reflect the accuracy of the $\text {NeurDNet}$ on classifying the “patients” or “tremor assessments” into PD or ET.

Upon fine-tuning the best hyper-parameters for the first-stage classifier through a rigorous grid-search procedure, a similar strategy is followed to identify the best hyper-parameters for the second-stage classifier. To develop the second-stage classifier, the first-stage is kept intact based on the best derived hyper-parameters and the second-stage classifier is updated to achieve the best performance, i.e., highest classification accuracy. The second-stage classifier can be characterized by two main hyper-parameters, i.e., the type of input features and the classification methodology. As the second-stage classifier is fed with the output of the first-stage block, the output of the first-stage classifier could be obtained either in binary format (class labels) or numeric format (probability associated with each class). The classification scheme of the second classifier is another hyper-parameter that its effect is investigated on the overall performance of $\text {NeurDNet}$. To this aim, we have employed a set of classifiers with different settings to be coupled with the first-stage classifier. The evaluated paradigms include RF, SVM, NB, LR, AB, LDA, QDA, DT, and MLP. Please note that the parameters defined in the parentheses of the first column of Table 2, indicate the option in which the classification algorithm is employed; “*Entropy*” and “*Gini*” define the clustering criteria for RF or DT, “*Radial Basis Function (RBF)*” and “*Linear*” define the type of kernel used by SVM, “*Singular Value Decomposition (SVD)*” and “*Least Squares Error (LSQR)*” indicate the eigenvalue solver for LDA, and “*MLP(N)*” defines a one layer neural network with *N* nodes.

Finally, to check the sensitivity of $\text {NeurDNet}$ to the amount of available training data and its capability to infer the underlying characteristics of the two diseases from the recordings, we have trained and evaluated the performance of network across different choices for test set population, which are 25%, 35%, 45%, 55%, 65%, and 75% of the whole dataset. Please note that the aforementioned ratio indicates the portion of dataset to form the test set. In addition, it should be noted that to decrease the effect of randomness in selecting the train/test subjects, we have performed each evaluation for 30 times and the mean accuracy of this comprehensive performance evaluation is reported in Table 2. As it is observed, the maximum classification accuracy is obtained when QDA classifier is coupled with the first-stage classifier, 75% of dataset is employed for training purposes, and probabilistic feature vectors are fed to the second-stage classifier. The second best accuracy is also obtained in similar settings, except for the case that 65% of dataset is employed for training. It is worth mentioning that the accuracy of the second-stage classifier is actually the accuracy of $\text {NeurDNet}$ in classifying the two diseases and is obtained by processing the whole tremor assessment of a subject, i.e., 54 tremor recordings from 6 tasks, in 3 trials, and in 3 channels.

### Architecture of $\text {NeurDNet}$

To differentiate between patients based on the type of their PHT, $\text {NeurDNet}$ takes advantage of a two-stage classification paradigm, which is designed to collectively employ the information stored in the time-series recordings of each patient, as well as their behavioral patterns in different tasks. Each tremor assessment consists of recordings in 3 channels from 6 tasks in 3 trials, which together add up to 54 single-channel tremor recordings. The first-stage classifier is designed to vote for each of the single-channel recordings, whether they are PD or ET. When 54 votes for a tremor assessment are collected, the class labels or probabilities associated with each class are fed to the second-stage classifier. We believe that the two stage classification paradigm enables us to extract the underlying and discriminating patterns of tremor signals as well as the discriminating behavioral patterns of patients in case of performing different tasks.

**Figure 3:**
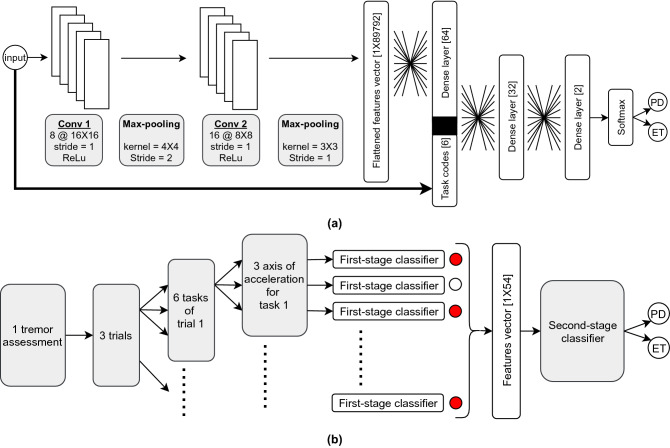
The overall processing framework of $\text {NeurDNet}$ to perform differential diagnosis between PD and ET. (**a**) This part depicts the processing pipeline for the first-stage classifier, which is based on convolutional neural networks. In this stage, a preliminary decision (PD or ET) is made on a single signal of tremor assessment, which is previously passed through the pre-processing block. This signal could be the acceleration of hand motion in any axis, from any task of any trial. (**b**) This figure shows the second stage of the classification process for each tremor assessment. In fact, each tremor assessment contains 54 tremor signals, where all of them are passed through the first-stage classifier. Then, the decision on each signal is aggregated in a vector of length 54 which forms the feature vector for the second-stage classifier.

The first-stage classifier takes advantage of convolutional neural architectures to process the spectrogram representations of the single-channel recordings. As shown in Fig. 3a, 2 convolutional layers followed by 3 dense layers build up the first-stage classifier. The details of the convolutional layers are given in the figure. The first dense layer employs ReLu activation functions and the second one employs Leaky–ReLu with the parameter of 0.1 as its activation function. A crucially important and novel characteristic of the designed first-stage classifier is employing shortcut bits for the second dense layer to introduce the origin of the input signal to the network. In other words, along with the spectrogram of a tremor signal, a binary vector of 6 bits is directly concatenated with the output of the first dense layer to form the input to the second dense layer. This vector encodes each clinical task with a binary vector and provides the network with extra information to conclude the label of a tremor signal. To train the network, the mean softmax cross entropy between the output of network and the true labels is minimized by employing Adam Optimizer with the learning rate of 0.0001. Performance monitoring over the validation set revealed that 44 epochs of training reach an optimal point in the learning curve and thus, we stop the training process after 44 epochs. The maximum accuracy achieved only on the first-stage classifier is $75.55\%$ over the validation set. It is worth noting that it is good practice to evaluate the framework only when the development phase is finalized and the whole processing framework ($\text {NeurDNet}$) is ready to be assessed on the test set. As such, for the first-stage classifier there is no choice other than reporting its performance over the validation set.

The second-stage classifier, on the other hand, is developed based on classical classification techniques and the maximum accuracy of 95.55% is achieved when Quadratic Discriminant Analysis (QDA) technique is applied on the outputs of the first-stage classifier. As shown in Fig. 3b, the votes of the first-stage classifier for one tremor assessment (54 votes for each tremor assessment) are collected in terms of probabilities for each class and a feature vector of length 54 is formed to train/evaluate the QDA classifier. To classify an unlabeled tremor assessment, the 54 features associated with it are derived to form the feature vector ${\varvec{f}}$. The classification is based on the prior probability of the classes given the feature vector, i.e., $p(y=class|{\varvec{f}})$, as such, according to the Bayes’ theorem, the posterior probability of $p({\varvec{f}}|y)$ needs to be calculated. In QDA classifier, the posterior probability is modeled as a multivariate Gaussian distribution, and thus, a likelihood ratio for the two classes given the feature vector and the information from training samples is calculated as

1 $$\begin{aligned} \text {Likelihood ratio}~=~\frac{\sqrt{2\pi {\varvec{\Sigma }}_{PD}}^{-1}~exp\Big (-0.5({\varvec{f}}- {\varvec{\mu }}_{PD})^T {\varvec{\Sigma }}_{PD}^{-1}({\varvec{f}}- {\varvec{\mu _{PD}}})\Big )}{\sqrt{2\pi {\varvec{\Sigma }}_{ET}}^{-1}~exp\Big (-0.5({\varvec{f}}- {\varvec{\mu }}_{ET})^T {\varvec{\Sigma }}_{ET}^{-1}({\varvec{f}}- {\varvec{\mu _{ET}}})\Big )}, \end{aligned}$$

where ${\varvec{\mu }}$ and ${\varvec{\Sigma }}$ respectively represent the mean and covariance matrix of features for the PD and ET classes.

## Results

In this section, the $\text {NeurDNet}$ framework is evaluated based on several different test paradigms and the results are presented. As thoroughly discussed in “Methods” section, the best classification accuracy of $\text {NeurDNet}$ is achieved when a CNN architecture, as shown in Fig. 3a, is used as the first-stage classifier and the outputs of the CNN model for each tremor assessment are fed to a quadratic discriminant analysis (QDA) model as the second-stage classifier, as shown in Fig. 3b. Each tremor assessment constitutes of 54 single-channel tremor signals and the role of the first-stage classifier is to classify each of these signals into PD or ET. Then, the collection of 54 predictions is fed to the QDA classifier as a feature vector, and the final vote for each tremor assessment is obtained by the second-stage classifier. It should be noted that the best classification accuracy, which according to Table 2 is $95.55\%$, is achieved when the training/test ratio of 3 : 1 ($75\%$ of data is reserved for training) is followed.

The results presented in Table 2 clearly suggest that the maximum classification accuracy is achieved when QDA classifier with probabilistic features are employed and the whole system is trained over $75\%$ of the dataset. In addition, it is worth highlighting that the consistency of results for different training/test ratios is also an important measure for robustness of a framework and reveals the capability of the $\text {NeurDNet}$ framework in generalizing over the underlying patterns of the studied phenomenon. Based on this argument, we can also nominate the Naive Bayes classifier as a successful classification method to be coupled with the first-stage classifier. The consistency of results for the NB classifier with probabilistic features across different training/test ratios, even for the minimum value of $25\%$ for training ($75\%$ for evaluation), reveals the superior capability of this classifier in grasping the overall distribution of features for the two PD and ET classes. The observed behaviour of the NB classifier in this work is also consistent with its renowned capability in extracting strong classification rules based on minimum amount of training data.

**Figure 4:**
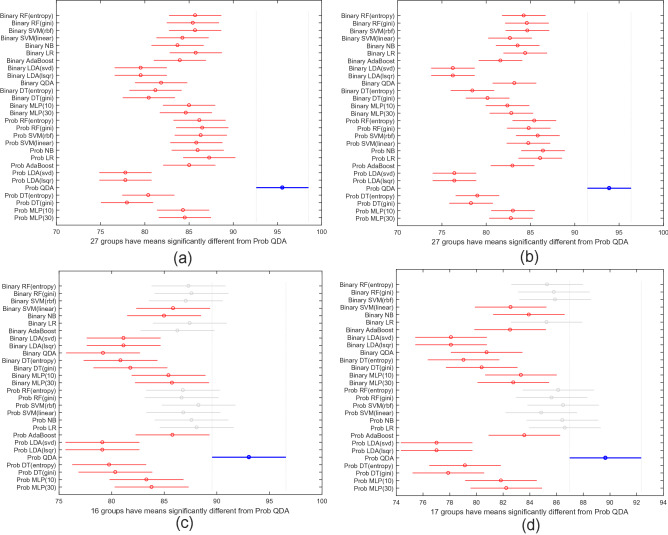
Results of the multiple comparison tests for the classification accuracy of NeurDNet across different scenarios. The term “Prob” in the vertical axis refers to the probabilistic features extracted from the first-stage classifier. The vertical axis represents different testing scenarios and the horizontal one represents the classification accuracy. Also note that the circles denote the mean classification accuracy and the lines define the range of the $95\%$ confidence interval. Please note that in all of the plots, the performance of **Prob QDA** (in blue) is compared with other scenarios. Any overlap between the lines of two scenarios corroborates that the performance of NeurDNet is not significantly altered by changing one hyper-parameter to another. The plots include significance tests for [portion of test set—patients’ visits accounted]: (**a**) $25\%$—all visits; (**b**) $35\%$—all visits; (**c**) $25\%$—first visits; (**d**) $35\%$—first visits.

To statistically compare the performance of NeurDNet across different scenarios, for each train/test ratio, we have performed a one-way analysis of variance (ANOVA) on the classification accuracy of the 30 random states obtained through different second-stage classifiers and for probabilistic/binary features. In other words, for each train/test ratio, the results of 28 scenarios (14 classifiers $\times$ 2 set of probabilistic/binary features) are statistically compared with each other through the ANOVA test. It is worth noting that prior to applying the ANOVA test, the normality of the obtained accuracies for each scenario is verified through Lilliefors test of normality^59^ with the confidence level of $5\%$. Afterward, the results are interpreted based on $95\%$ confidence criteria by multiple comparison plots, as shown in Fig. 4. It is worth highlighting that in the comparisons plotted in Fig. 4, the circle denotes the average of classification accuracies in each scenario, and the lines denote the range for which the confidence interval is defined. In other words, if two cases have overlapping lines, it is understood that the classification accuracies for the two cases are not statistically ($95\%$ confidence) different. The opposite of this statement also holds for non-overlapping lines, which indicates statistically different accuracies for two scenarios. It is worth highlighting that in the multiple comparison plots shown in Fig. 4, the vertical axis denotes the 28 different evaluation scenarios, while the horizontal axis represents the classification accuracy. As can be understood from Fig. 4a,b, the first-stage classifier coupled with the QDA classifier and with probabilistic features offers statistically significant better results compared to its counterparts, when $25\%$ and $35\%$ of the dataset populate the test set, respectively.

**Figure 5:**
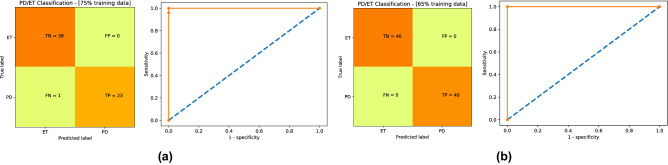
Confusion matrix and the ROC diagrams associated with the 2 winning frameworks for PD/ET classification. Please note that AUC stands for area under curve. Two winning paradigms of $\text {NeurDNet}$ are when QDA classifiers is coupled with the first-stage classifier and (**a**) $75\%$ and (**b**) $65\%$ of the dataset is used for training process, respectively.

To further investigate the performance of $\text {NeurDNet}$, we derived the confusion matrix and the receiver operating characteristics (ROC) curve for the winning frameworks of $\text {NeurDNet}$. ROC curve helps us understand the diagnostic capability of a binary classifier by measuring the sensitivity and specificity of classification for different thresholds of distinguishing the two diseases. To define the meaning of sensitivity and specificity in this context, we first need to define the terms of “positive” and “negative” diagnosis. Basically, the term “negative” stands for healthy diagnosis of an individual and the term “positive” stands for the opposite. However, as in this work we are not dealing with a healthy/patient problem and our goal is to distinguish between the two diseases, we redefine the terms “positive” and “negative” as being classified as PD and ET, respectively. Thus, the sensitivity (specificity) of $\text {NeurDNet}$ is the ratio of the correct PD (ET) classifications over the total number of PD (ET) cases. ROC curve illustrates *sensitivity* against $(1-specificity)$ and helps physicians to choose a proper threshold to attain a certain degree of sensitivity or specificity. In addition to determining the classification threshold, another important classification measure that is derived based on the ROC curve is the “area under the curve (AUC)” criteria. AUC indicates how well a classifier distinguishes two classes and its value in the range between 0.5 to 1 reflects the performance of the classifier from “no discrimination capacity” to “perfect discrimination capacity”, respectively. To obtain the confusion matrix and ROC curves for $\text {NeurDNet}$, the two most accurate classification paradigms in Table 2 are selected and the results are shown in Fig. 5a,b. It is worth mentioning that to generate the plots in Fig. 5, we need to analyze the output of a complete classification pipeline with fixed training and testing set, however, the reported values in Table 2 are obtained by averaging over 30 trials, thus the mean value is not necessarily associated with any of the 30 random runs. To generate the plots in Fig. 5a,b, we used the training set that leads to maximum classification accuracy among the 30 random formations of the train and test sets.

Since some of the patients affected by either PD or ET show bilateral PHT, and due to the need imposed by the data collection protocol that the hands showing PHT must be evaluated and recorded, this dataset includes both unilateral and bilateral recordings from different patients. It is worth discussing that the existence of unilateral and bilateral recordings in one dataset might raise some questions on the leakage of information from training set to test set, i.e., in bilateral cases, splitting the recordings of two hands into the training set and the test set. However, we would like to highlight the fact that in this work, the formation of train and test sets is solely based on the patients, rendering zero chance for the leakage of information from training set to the test set. For the bilateral cases that exist in the test set, the final decision for each patient is obtained by performing a logical AND on the decision of the NeurDNet on the tremor assessments from the two hands, meaning that the patient is correctly classified if and only if the two decisions are correct.

### Explainability of $\text {NeurDNet}$

**Figure 6:**
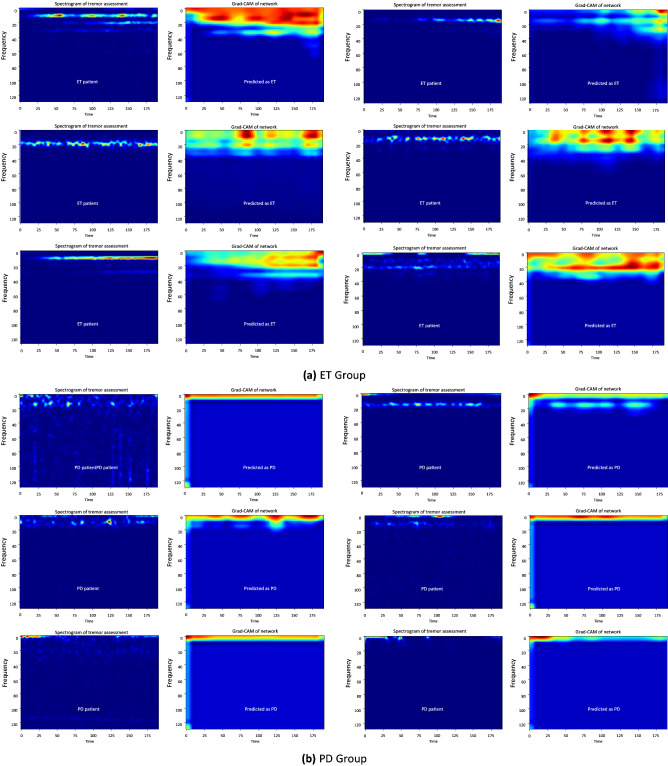
Analysis of explainability for $\text {NeurDNet}$. It should be highlighted to convert the values *y-axis* scale to frequency in Hz, the values need to be multiplied by 100/256.

Generally speaking, the capability to identify and explain the internal process that leads to a certain outcome is referred to as the *explainability* of machine learning models (XAI), which plays an important role in approving the applicability of model and reliability of its results. When it comes to employing deep neural networks in biomedical domain, due to the sensitivity of application and the risk of fatal errors, the explainability of the model becomes of much greater importance. In this subsection, we investigate the explainability of $\text {NeurDNet}$ by extracting clues in the tremor signals that are important and noticeable in concluding the label of an unseen tremor assessment. In other words, we discover the regions in the spectrotemporal representation of tremor recordings, which motivate the network to select one class over the other. To this aim, the “Gradient-weighted Class Activation Mapping (Grad-CAM)^60^” methodology is employed to discover the parts of the input spectrogram to the CNN, which contribute to assignment of a label to the input. To obtain the Grad-CAM representations of $\text {NeurDNet}$, as shown in Fig. 6, all the output nodes of the first-stage classifier are set equal to zero except the one that corresponds to the correct label. Then, the gradients of this output are backpropagated to the network and a heatmap mask for the input signal is obtained. The mask assigns a weight to each pixel of the input signal to determine its importance in concluding the final label. To implement this process, we have employed the “keras-visualizations” library^61^ in Python language.

**Figure 7:**
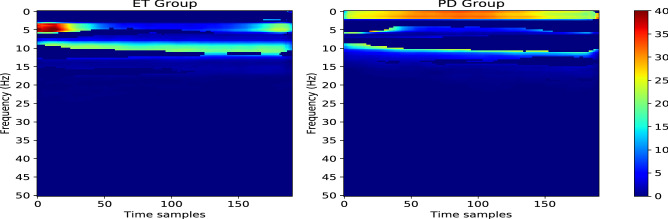
Results of the statistical test over the Grad-CAM analysis of $\text {NeurDNet}$ for the two diseases. The intensity of different parts in the spectrogram determines the importance of the region for $\text {NeurDNet}$ to conclude the class of the tremor assessment.

Although the Grad-CAM plots in Fig. 6 clearly represent the important regions for $\text {NeurDNet}$ to distinguish PD from ET and provide some interpretable insights on the valuable spectral contents for each of the two diseases, we need to statistically validate our observations over the whole dataset and investigate if the observed differences are meaningfully valid for all of the samples in the dataset. To this aim, we statistically and in a pixel-wise fashion compare the heatmaps for the two classes to check if any pixel takes significantly different values for the two classes. In this regard, first, we derived the Grad-CAM representation of $\text {NeurDNet}$ for all of the PD and ET tremor assessments that are correctly classified. Due to the large population of instances for each group (3788 and 2473 for ET and PD, respectively), the “$z-test$” needs to be employed to check if the mean of the Grad-CAM analysis for each pixel and of the two groups is significantly different. As the $z-test$ procedure is based on normal distribution of data, we first performed the D’Agostino and Pearson’s test^62^ to verify the normality of data. Once this condition was relaxed, which was expected due to the large number of instances and insights from the central limit theorem, we derived the element-wise $z\, score$ across the whole dataset and between the two groups as follows

2 $$\begin{aligned} z = \frac{{\bar{g}}_{ET} - {\bar{g}}_{PD}}{\sqrt{\frac{\sigma _{ET}^2}{n_{ET}} +\frac{\sigma _{PD}^2}{n_{PD}}}}, \end{aligned}$$

where ${\bar{g}}_{ET}, {\bar{g}}_{PD}, \sigma _{ET}^2, \sigma _{PD}^2, n_{ET},$ and $n_{PD}$ are the mean value of Grad-CAM pixel for ET group, mean value of Grad-CAM pixel for PD group, variance of the pixel across ET group, variance of the pixel across PD group, population of ET samples, and population of PD samples, respectively. Please note that $z\, score$ is calculated for each pixel across the two groups and the $p\,value$ is respectively obtained for each pixel. According to the formulation of $z\, score$ in Eq. (2), positive $z\, score$ corresponds to higher attention of $\text {NeurDNet}$ to ET features and the opposite stands for the PD group. Thus, to derive the masks associated with each group, we obtain the area under a standard normal distribution, $auc(z_0)$, given by

3 $$\begin{aligned} auc(z_0) = \int _{-\inf }^{z_0}p_Z(z) dz, \end{aligned}$$

where $Z \sim {\mathscr {N}}(0,1)$ or in other words $p_Z(z) = \frac{1}{2\pi } \exp (-z^2/2)$. For the ET group, the mask is of the same dimension as the input spectrogram and is a zero matrix, except for the pixels that $auc(z) > 0.99$. Conversely, for the PD group, the mask is obtained by selecting the pixels for which the $auc(z)<0.01$. In fact, this process is equal to selecting the pixels where the Grad-CAM analysis of $\text {NeurDNet}$ shows significantly different means for the two groups by setting $\alpha = 0.02$ ($p\,value < 0.02$). Afterwards, the masks are applied on the mean Grad-CAM representation of $\text {NeurDNet}$ for PD and ET groups to reveal the important temporal and spectral regions for classification of each group. The results of this analysis are shown in Fig. 7.

The plots in Fig. 7 clearly represent the informative regions in the spectrotemporal plots of tremor signals, which are insightful for the differentiation of Parkinsonian tremor from ET. As it is observed, PD is mainly characterized by occurrence of low frequency vibrations on the hand motion, whereas ET is mainly characterized by high spectral activity in the hand motion signals. The highlighted regions for each disease are also compatible with their physiological characteristic, where ET is known to occur in a wider spectral range than PD. It should be noted that the highlighted regions in the mean spectrotemporal maps of ET and PD populations in Fig. 7 do not imply that the spectral contents of each disease are only stored in those areas. On the contrary, the highlighted regions identify statistically significant regions in the spectrotemporal map of signals, which provide informative and strong clues for the network and potentially for the physicians to discriminate the two diseases.

### Analysis of the dominant features of the second-stage classifier

**Figure 8:**
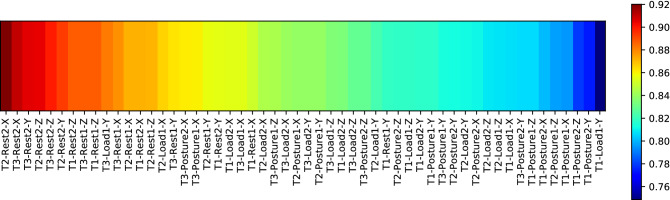
Results of sequential feature selection for the features that are fed to the second-stage classifier. Please note that these results are obtained through a 5-fold cross-validation process, when $75\%$ of dataset is used for training. It should be highlighted that in this analysis, the probabilistic features due to their superior performance over binary features are employed, and the label of each feature is formed as [TrialNumber-TaskName-RecordingChannel].

Similar to the previous subsection where we investigated the learned features of the first-stage classifier through the Grad-CAM analysis, in this subsection, we identify the importance of task-specific features to classify the tremor assessments in the second-stage classifier. The results presented in this subsection are obtained by analyzing the winning architecture of $\text {NeurDNet}$, which is trained over $75\%$ of the dataset and employs probabilistic features with QDA classifier. To identify the role of each feature in forming the final decision of $\text {NeurDNet}$ for an input signal, a sequential and iterative feature selection approach, referred to as the wrapper method, is employed. In this technique, the classification accuracy for different subsets of features is calculated and the subset with the highest classification accuracy contains the most influential features. In addition, the wrapper method does not utilize similarity or scoring criteria to compare the features with labels; instead, the dominant features are selected based on their effect in the final classification accuracy. In this work, to discover the efficacy of each feature, the best feature through the discussed sequential process is selected, then it is removed from the pool of features, and then again the best feature in the pool is selected. This process continues until all of the features are drawn from the pool and all of the features are sorted based on their role in forming the final decision of the classifier. The results of this process are shown in Fig. 8.

The plot in Fig. 8 determines the efficacy of each tremor assessment task in providing useful information for differentiating of PD from ET, and reveals that the features obtained from the “Rest1” and “Rest2” tasks convey exceptionally valuable information to the classifier to discriminate between the two diseases. In fact, the plot shows the achieved classification accuracy when only one of the features is utilized to form the classifier. As we have a pool of 54 features for each tremor assessment, investigating the efficacy of all possible subsets of features on the classification accuracy of $\text {NeurDNet}$ would have imposed high computational burdens on the development phase (assessment of $2^{54}$ cases) and thus, we have examined the effect of features based on a naive assumption where features are considered independently.

### Accuracy of the first-visit diagnosis

Another characteristic of $\text {NeurDNet}$, which is crucially important from a clinical point of view, is the accuracy of $\text {NeurDNet}$ in classifying patients during their first visit to the clinic. As discussed earlier, the majority of patients in this study (45 out of 47 in the PD group and 31 out of 34 in the ET group) have participated twice in the data collection phase, where there is an interval of 6 weeks between the two visits. Until now, the reported performances of $\text {NeurDNet}$ are based on collectively processing the tremor assessments from the first and the second visits, which might be biased due to presence of any identifiable or unidentifiable role playing factor between the two visits. In other words, some factors like the familiarity of patients with the tasks, and the effect of any potentially received medication within the 6 weeks period can change the distribution of input data between the two visits, and may leave a positive or negative impact on the performance of $\text {NeurDNet}$. The ability to differentiate the PHT caused by PD from the one caused by ET, when the differences are subtle, as early as possible in disease progress (ideally the first visit), can provide imperative assistive information for tremor management and potentially evaluating the type and severity of the condition. In addition, the response of patients to tremor management strategies (e.g., medication) should be considered as part of this differentiation. The results of this analysis are provided in Table 3. The presented results confirm that the $\text {NeurDNet}$ framework achieves a high classification accuracy over the tremor assessments recorded during the first visit of patients to the clinic. As it is understood from the results, the maximum classification accuracy of $93.05\%$ is achieved when QDA classifier is coupled with the first-stage classifier and $25\%$ of dataset is reserved for evaluation. It is worth reiterating that the formation of the training and evaluation sets is based on the subjects and all of the tremor assessments from one subject contribute only to one set, even if the patient has revisited the clinic in 6 weeks. Another point with regards to Table 3 is that the reported classification accuracies are obtained via a Monte Carlo simulation technique, i.e., averaging the classification accuracy of $\text {NeurDNet}$ over 30 random formations of the training/evaluation sets. By revisiting the plots in Fig. 4c,d, it is understood that despite the fact that employing the QDA classifier with probabilistic features as the second-stage classifier partially beats all of the other scenarios, it still offers the best mean classification accuracy among others, which approves the superior performance of NeurDNet, when QDA classifier is coupled with the first-stage classifier through probabilistic features.

## Discussion

One important aspect of the growing literature on the problem of PD-ET classification is the role that different machine learning techniques play in structuring the processing pipelines. Broadly speaking, the utilization of DL techniques can be seen as a major game changing aspect of the research work published on this topic. The works based on classical ML (non-DL) techniques mainly includes a feature extraction/generation block that requires researchers to come up with informative features to discriminate PD from ET. Engineering such features often requires some assumptions on the characteristics of the two diseases, which may limit the generalization of the model. Despite the drawbacks mentioned with the feature generation phase, such techniques require less amount of training/evaluation data, which is a crucially important feature for medical datasets with limited sizes. On the other hand, the DL-based methods bypass the manual feature extraction/generation phase by automatically inferring discriminating clues in the input data through observing numerous training examples. Although this approach requires extensive datasets to train and evaluate the model, they often lead to higher classification accuracy and better generalization over the diverse range of characteristics in and among patients. This comparison can also be partially observed in NeurDNet, where the first-stage classifier takes advantage of a DL-based pipeline and the second-stage classifier is a classical ML technique (QDA classifier). In fact, to develop the first-stage classifier, an abundance of training samples (250 tremor assessments $\times$ 54 tremor signals = 13, 500) is available, which enables us to employ DL methods. However, for the second-stage classifier, since only 81 patients are studied, a classical ML technique is utilized. It is worth highlighting that both of the classifiers in the two stages are properly backed with statistical significance tests and validation results for their generalization over the problem in hand.

Next, we provide detailed discussions on the novelties of the $\text {NeurDNet}$, comparisons with prior works, and its advantages and disadvantages:

- Novel features of the $\text {NeurDNet}$ framework:
  - $\text {NeurDNet}$ produces a novel and accurate machine intelligence pipeline designed on a particular collection of hand tremor to conduct a differential diagnosis between PD and ET.
  - Using the state-of-the-art Grad-CAM analysis, this paper, for the first time, highlights segments of the spectrotemporal behavior of hand tremors, which have the most discriminative power for differentiating PD from ET using advanced recurrent neural network approaches. This makes the proposed information processing pipeline an explainable model (under XAI), which is the new generation of machine intelligence, tangential to the conventional black-box implementation, which does not provide any insight on the decisions made and was susceptible to biases in the datasets. This major novelty of the proposed $\text {NeurDNet}$ is highlighted as the comprehensive study and analysis over the explainability of network and the corresponding statistical analysis conducted in the paper over the clues in the input signals leading to certain labels (PD or ET). The results of this analysis not only provide clinically viable information on the clues to discriminate PD from ET, but also relax the concerns on learning the structural and unwanted biases in the input data that can take part in discriminating the two diseases.
  - $\text {NeurDNet}$ introduces a sequential processing pipeline based on a CNN core and a QDA classifier, which offers a multi-stage classification paradigm for differentiation of PD from ET. This unique architecture enhances the reliability of the system in determining if the unseen patients are PD or ET by analyzing the dynamics of hand in a hierarchical format.
  - The processing framework of $\text {NeurDNet}$ is intelligently designed to maximize the amount of information exploited from the dataset by not only processing the signals representing the dynamics of hand motion but also incorporating the task labels to further assist the framework in interpreting the signals. This novelty in the architecture of the neural network catalyzes the classification accuracy of $\text {NeurDNet}$.
  - The $\text {NeurDNet}$ framework is developed over a substantially large dataset of hand dynamics containing 87.5 h of PHT recordings from 81 PD and ET patients. In this unique dataset, the dynamics of hand motion are examined in 7 different scenarios, which further increase the amount of information obtained from limb movement in patients with PD or ET.
- Comparison with prior works: By revisiting the list of recent research works with potentially relevant objectives as $\text {NeurDNet}$ in Table 1, it is readily understood that $\text {NeurDNet}$ outperforms the state-of-the-art accuracy in discriminating PD from ET. It also offers a novel machine intelligence pipeline which can be interpreted from the clinical point of view. Considering the predecessors of $\text {NeurDNet}$ with the highest classification accuracies (before the invention of NeurDNET in this paper), i.e., References^15,49,52–54,63^, it is understood that $\text {NeurDNet}$ not only excels the classification accuracy of the research that is based on accelerometer data but also outperforms the one based on Electromyogram (EMG) signals recorded from a tremorous hand (which was supposed to have richer neurophysiological content in the signal). To be more specific, here we provide an itemized comparison with recent research publications, leading the state-of-the-art classification accuracy for discriminating PD from ET.
  - The work by Di et al.^15^ has collected accelerometer data and offers a classification accuracy of $92\%$. This work uses a tremor stability index as the feature for classification of PD from ET, which is derived by performing spectral analysis over a signal of length 100 s. Through their experiments, a certain threshold value for the stability index is determined for classification. On the contrary, the classification strategy of the $\text {NeurDNet}$ assigns a probability to the final label of an unseen patient by analyzing the acceleration of hand motion in different axes, different tasks, and different trials, which offers a higher classification accuracy and a much more robust diagnostic framework. In fact, the proposed strategy enhances the reliability of the system in classifying patients and grants it a great degree of generalization over the characteristics of hand tremor. Besides the fact that our proposed $\text {NeurDNet}$ framework achieves a higher classification accuracy, we believe that our analysis over 87.5 h of tremor recordings (compared to 2.527 h in reference^15^) achieves a better generalization over the wide and overlapping range of features in hand tremor among PD and ET patients, and provides a more robust classification paradigm.
  - Another counterpart to the $\text {NeurDNet}$ that achieved the state-of-the-art classification accuracy of $94\%$^49^ employs a combination of EMG recordings from tremorous hand and a set of physiological features collected from 54 patients and introduces a classification framework based on decision trees. Comparing the devices, it can be mentioned that EMG studies are typically more complex, requires more rigorous calibration, and is more expensive, all of which would affect the scalability of the machine intelligence in clinics, especially for those who are not sophisticated. Our proposed $\text {NeurDNet}$ framework not only has recruited more number of patients for its analysis, which leads to better generalization over the inter and intra-personal variance of features in the hand tremor but also only employs an accelerometer to collect the required signals, which is a more cost-effective, more scalable, and portable solution, and requires a very each calibration process when compared with EMG studies.
  - Technically speaking, the framework proposed by Locatelli et al.^52^ is developed based on several features extracted from the Power Spectral Density (PSD) of the tremor signals by fitting a bell-shaped function to their PSD. However, one of the main sources of ambiguity in differentiating between PD and ET is the overlap in their spectral contents. Moreover, it is known that fatigue in the tremorous muscles and the emotional state of the patient can potentially influence the characteristics (spectral contents) of tremor. On the other hand, the decisions in NeurDNet are derived by evaluating the patient in 6 tasks (different postures) and 3 trials (effect of fatigue), and fusing the information extracted from the spectrograms of tremor signals with the type of tasks from which the signals are recorded. In other words, NeurDNet can potentially offer better generalization and robustness in decision making by incorporating several sources of information. Moreover, the framework proposed in reference^52^ is developed based on a dataset recorded from 24 patients including 7 ET cases and 17 PD patients. NeurDNet, on the other hand, employs a dataset of 81 patients for training and evaluation purposes. The generalization of an AI-based model over a problem is proportionally tied to the size of the dataset, and thus, a better generalization is expected for NeurDNet. In addition, the employed dataset in reference^52^ results in an imbalance of data for the PD and ET classes. More specifically, the proportion of PD and ET patients to the size of dataset in this work is $58\%$ and $42\%$ respectively, while in reference^52^, the proportions are $71\%$ and $29\%$. The imbalance of the dataset, especially towards the ET group, can potentially introduce biases to the system, which reduces the reliability of the decisions. To be more specific, the performance of the framework introduced in the reference^52^ is measured based on 5-fold cross validation, which according to the number of ET patients (7 cases), means that in 2, folds there are 2 ET patients in the test set, and in 3 folds, there is only 1 ET patient in the test set. Based on this argument, we believe that the reported accuracy of the framework in reference^52^ ($95.8\%$), although numerically comparable with the one for NeurDNet, falls far behind NeurDNet in terms of reliability and generalization.
  - The work by Moon et al.^53^ presents a set of processing techniques to address the problem of PD-ET classification based on several hand-crafted features extracted from motion signals recorded from patients with PD and ET. Although the size of the employed dataset is much larger than the one for NeurDNet (567 patients compared to 81 patients), there is a tremendous imbalance between the PD cases ($n=524$) and ET cases ($n=43$) in the article. To tackle this imbalance, the authors have employed a synthetic minority oversampling technique (SMOTE), which produces synthetic data for the class with fewer instances (ET class in this paper). Since the synthetic samples will represent the same characteristics of the original ET samples, it is doubtful there is any added value in terms of generalization over the characteristics of the essential tremor. In fact, we believe that using synthesized samples is somehow equivalent to leakage of information from the training set into the test set, which eventually may result in providing misleading information about performance for the model. Besides, the proposed methodology in reference^53^ requires several inertial units to be mounted on the body of patients, which requires medical-grade acquisition devices. However, NeurDNet is developed based on inertial measurements from on accelerometer mounted on the dorsum of hand. In other words, NeurDNet offers a more cost-effective solution, which paves the way for utilization of wearable and commercial sensors for the purpose of diagnosis. Finally, it is worth highlighting that the overall classification accuracy of the NeurDNet ($95.55\%$) is higher than the best accuracy reported in reference^53^ ($92\%$).
  - The work by Duque et al.^54^ introduces a processing framework to differentiate between PD from ET based spectral features manually extracted from accelerometer data. Spectral-based features are prone to large variations in and across different patients due to influencing factors such as fatigue in tremorous muscles, and the emotional state of the patient. In comparison, NeurDNet obtains a final decision on a patient by processing 54 spectrograms (temporal and spectral features) of tremor signals derived by evaluating the patient in 3 trials (different levels of fatigue), 6 tasks (different postures), and 3 different axes. Moreover, although the data collection in reference^54^ is performed by the built-in accelerometer of a mobile phone, which can potentially offer a cost-effective and accessible solution, there are potential issues regarding generalization of the introduced model over the wide range of characteristics in Parkinsonian and Essential tremor. To be more specific, by considering the tabulated performance results in this article, a huge variation between the average performance and the best performance is observed ($\ge 20\%$), which does not assure a robust and reliable generalization over the training set. Besides, the best classification accuracy between PD and ET in this work is $84.4\%$, while for NeurDNet, it is $95.55\%$.
  - Although the work by Nanda et al.^63^ aims at discriminating between PD from ET based on accelerometer signals, the approach employed is fundamentally different from NeurDNet, and we do not think that these two approaches can be considered under the same category. In fact, the dataset of this work includes accelerometer recordings from only 2 patients (1 PD and 1 ET), and the classification is actually the process of learning from some segments, and labeling some other segments of the signals. However, the NeurDNet is developed to classify different patients into the PD and ET classes. Technically speaking, reference^63^ employs Wavelet transformations to decompose the tremor signals into several spectra-temporal components for subsequent feature engineering processing blocks. Since the sampling frequency of the signals in reference^63^ is 1000 Hz, the components extracted through Wavelet transforms are specific to certain frequency bands, i.e., [500–250] Hz, [250–125] Hz, [125–62.5] Hz, $\ldots$ . Decomposition components in these coarse ranges might not be able to fully detect and distinguish the specific spectral contents of PD and ET. In addition, the analysis of explainability over the wavelet components might not fully correspond to the physiological background of PD and ET.
- Advantages and disadvantages of $\text {NeurDNet}$: Besides the fact that our proposed $\text {NeurDNet}$ framework achieves a higher classification accuracy, we believe that our analysis over 87.5 h of tremor recordings (compared to 2.527 h in reference^15^) achieves a better generalization over the wide and overlapping range of features in hand tremor among PD and ET patients, and provides a more robust classification paradigm. Also, our employed dataset examines the dynamics of hand in 6 different static positions, which further reveals the behavioral patterns of the hand tremor and minimizes the risk of overfitting in the framework. In addition, the $\text {NeurDNet}$ is grounded on analyzing the accelerometer signals, representing the dynamics of hand motion in different axes, which compared to a considerable number of research works focused on differentiating PD from ET by means of EMG signals, offers a more cost-effective, accessible, and portable solution. It is worth highlighting that although the proposed $\text {NeurDNet}$ requires a larger data collection from each patient, which might be tedious or boring for some patients, given the importance of correct diagnosis and the consequences associated with misdiagnosis of patients, we believe that $\text {NeurDNet}$ is a more robust and reliable classification paradigm for the PD vs. ET problem. Above all, the $\text {NeurDNet}$, for the first time in this domain, presents a unique and comprehensive study of the explainability of the classification model, which is supported by a thorough statistical analysis of the results. This important feature, not only provides viable and statistically significant information for clinicians to discriminate PD from ET but also relaxes the concerns on the curse of overfitting to biases in the analyzed signals.

As a final note to our discussion, it is worth comparing the $\text {NeurDNet}$ with our previously developed PHTNet framework^25^. Below, we provide a point-by-point comparison between the two works:

- Rationale: It should be noted that the objectives and rationales for the two works are completely different and distinct from each other. The PHTNet is a tremor estimation designed to be used for mechanical compensation using robotic rehabilitation systems; however, the submitted $\text {NeurDNet}$ is a diagnosis framework designed to conduct differentiative diagnosis between PD and ET. The output of the PHTNet is “a signal representing the involuntary component of hand motion” in patients with pathological hand tremors, while the output of the $\text {NeurDNet}$ is a diagnostic label denoting whether the studied patient has developed Parkinsonian tremor or ET. The working hypothesis of the PHTNET was that using denoising advanced machine learning approaches, the future episodes of hand tremors can be estimated and separated from the voluntary (though high-frequency) component of hand motion, minimizing the time latency, which is a major concern when robotic systems are used to compensate for the hand tremor. In the PHTNet, the data gathered from two groups of patients are collectively processed to generate a Recurrent Neural Network (RNN) approach as an intelligent filter. The working hypothesis of the submitted $\text {NeurDNet}$ is that machine learning approaches can be used to differentiate PD from ET when the tremor is collected from a particular systematic study protocol stimulating different synergistic muscle contraction, as suggested in this paper. The $\text {NeurDNet}$ is a diagnostic procedure, a data-driven framework based on Convolutional Neural Networks (CNN) to discriminate between the two groups of PD and ET patients. In other words, in the submitted work, PD and ET patients are compared with respect to each other to highlight their differences.
- Datasets, device, and patients: Both $\text {NeurDNet}$ and PHTNET are under the umbrella of a very larger clinical study and project; thus, the two papers partially share the dataset from a larger poll of patients collected by the very accurate technology available at Prof. Jog’s clinic. It should also be noted that the larger study has other angles and clinical measurements that have not been included/studied in these two works.
- Analysis paradigms: As pointed out earlier, PHTNet addresses the problem of estimating and predicting the involuntary component of hand motion in patients with PD and ET by means of RNNs as part of our robotic project, which aims to cancel out hand tremor using active robots which require very low latency of tremor estimation. $\text {NeurDNet}$, on the other hand, proposes a hybrid architecture based on CNNs and Quadratic Discriminant Analysis (QDA) to classify patients into PD or ET and conduct an intelligent differentiation. It is worth highlighting that not only the processing (analysis) paradigm of the two studies are completely different but also nature, type, implementation, validation, and outputs are different.

## Data Availability

The datasets generated and/or analyzed during the current study are not publicly available due to the confidentiality restrictions imposed by the approved ethics of study but are available from the corresponding author on reasonable request.
